# An Effort to Use Human-Based Exome Capture Methods to Analyze Chimpanzee and Macaque Exomes

**DOI:** 10.1371/journal.pone.0040637

**Published:** 2012-07-27

**Authors:** Xin Jin, Mingze He, Betsy Ferguson, Yuhuan Meng, Limei Ouyang, Jingjing Ren, Thomas Mailund, Fei Sun, Liangdan Sun, Juan Shen, Min Zhuo, Li Song, Jufang Wang, Fei Ling, Yuqi Zhu, Christina Hvilsom, Hans Siegismund, Xiaoming Liu, Zhuolin Gong, Fang Ji, Xinzhong Wang, Boqing Liu, Yu Zhang, Jianguo Hou, Jing Wang, Hua Zhao, Yanyi Wang, Xiaodong Fang, Guojie Zhang, Jian Wang, Xuejun Zhang, Mikkel H. Schierup, Hongli Du, Jun Wang, Xiaoning Wang

**Affiliations:** 1 School of Bioscience and Bioengineering, South China University of Technology, Guangzhou, China; 2 BGI-Shenzhen, Shenzhen, China; 3 Primate Genetics Program, Oregon National Primate Research Center, Oregon Health and Sciences University, Beaverton, Oregon, United States of America; 4 School of Biological Science and Medical Engineering, Southeast University, Nanjing, China; 5 Bioinformatics Research Center, Aarhus University, Aarhus C, Denmark; 6 Institute of Dermatology and Department of Dermatology, No.1 Hospital, Anhui Medical University, Hefei, Anhui, China; 7 State Key Laboratory Incubation Base of Dermatology, Ministry of National Science and Technology, Hefei, Anhui, China; 8 Science and Conservation, Copenhagen Zoo, Frederiksberg, Denmark; 9 South-China Primate Research and Development Center, Guangdong Entomological Institute, Guangzhou, China; 10 Guangdong Laboratory Animals Monitoring Institute, Guangzhou, China; 11 Bioinformatics, Department of Biology, University of Copenhagen, Copenhagen, Denmark; 12 Chinese PLA General Hospital, Beijing, China; Tulane University, United States of America

## Abstract

Non-human primates have emerged as an important resource for the study of human disease and evolution. The characterization of genomic variation between and within non-human primate species could advance the development of genetically defined non-human primate disease models. However, non-human primate specific reagents that would expedite such research, such as exon-capture tools, are lacking. We evaluated the efficiency of using a human exome capture design for the selective enrichment of exonic regions of non-human primates. We compared the exon sequence recovery in nine chimpanzees, two crab-eating macaques and eight Japanese macaques. Over 91% of the target regions were captured in the non-human primate samples, although the specificity of the capture decreased as evolutionary divergence from humans increased. Both intra-specific and inter-specific DNA variants were identified; Sanger-based resequencing validated 85.4% of 41 randomly selected SNPs. Among the short indels identified, a majority (54.6%–77.3%) of the variants resulted in a change of 3 base pairs, consistent with expectations for a selection against frame shift mutations. Taken together, these findings indicate that use of a human design exon-capture array can provide efficient enrichment of non-human primate gene regions. Accordingly, use of the human exon-capture methods provides an attractive, cost-effective approach for the comparative analysis of non-human primate genomes, including gene-based DNA variant discovery.

## Introduction

Non-human primates are increasingly studied as highly relevant animal models for human biomedical diseases and disorders. Members of the *Macaca* genus are among the most commonly studied non-human primates, due to their close evolutionary relationship to humans, analogous disease susceptibilities, and wide-spread commercial availability. The rhesus macaque (*Macaca mulatta),* estimated to have shared a common ancestor with humans approximately 25 million years ago (MYA) [1], is one of the most widely studied macaques. Genetic studies have shown the rhesus macaque to have common genetic risk factors with humans for age-related macular degeneration [2] behavioral disorders [3], [4]_ENREF_4 and reproductive disorders such as amennorhea [5]. A close relative of the rhesus macaque, the Japanese macaque *(M. fuscata)* has served as a model for multiple sclerosis [6] and ischemia [7], [8]_ENREF_8. The crab-eating or cynomolgus macaque *(M. fascicularis)* is widely used in studies of amyotrophic lateral sclerosis [9], and depression [10], among other disorders.

The chimpanzee (*Pan troglodytes*), is more closely related to humans than the macaques, sharing a common ancestor approximately 5–7 MYA [1]. The more recent divergence between humans and the chimpanzee has been of particular importance to the study of human evolution and speciation [11], [12]_ENREF_12. In the field of comparative genomics, the chimpanzee genome provides a critical insight into studies of positive selection in primate genomes [13]. The chimpanzee has also served as a important model for neuroscience research, including studies of cognition [14], neurobiology [15], and behavior [16].

With the recent advance in genomic technologies, interest in comparative analysis of non-human primates, particularly as they relate to biomedical and evolutionary studies, has been rapidly expanding [17], [18]. However, such studies are limited by the financial costs, computational requirements and effort required to generate genome-wide variant data on a large scale. Although improvements in next-generation sequencing (NGS) technology have already sharply reduced the cost of sequencing, the non-human primate still significantly lags behind in the comprehensive characterization of genome variation.

Exome sequencing has proven to be a powerful and efficient approach in human genetics studies [19], as it allows an unbiased investigation of almost all protein-coding regions in a large sample of individuals, at a fraction of the cost of whole genome sequencing. The method has been successfully applied to causative gene identification of several rare monogenic diseases such as Miller syndrome [20] spinocerebellar ataxias [21] and retinitis pigementosa [22]. A study of 50 Tibetan exomes uncovered a number of high-altitude adaptation related genes [23]. If the human exome-capture tools can be applied to the closely related non-human primate species, it could provide an opportunity to efficiently advance the pace of discovery of non-human primate sequence variants.

The human and chimpanzee genomes are about 99% identical, while macaques and human genomes are an estimated 93% conserved [17], [18]. Given the high level of sequence conservation for coding regions among primates, we considered whether it would be feasible to efficiently enrich the exonic sequences of primate species using human-based exon capture designs. Applying exon-capture technology to non-human primate research would not only minimize cost, but it would also reduce the computational effort required for deep sequence analysis. Importantly, exome-sequencing approaches would expedite the discovery sequence variants of greatest interest to many investigators, those located in gene coding regions.

Similar efforts have been used to successfully enrich and sequence target regions of the Neanderthal genome [24]. More than a megabase of captured sequence was recovered from Neanderthal DNA, despite DNA degradation and the presence of significant microbial DNA contamination. This achievement provides support for the use of human exon-capture reagents for the study of more distantly related human ancestors.

Here we report an effort to use human based exome capture to analyze chimpanzee and macaque exomes. Nineteen non-human primates, involving 3 species, were evaluated. We report the utility of the human exon array tool for exon enrichment, DNA variant discovery, and for comparative genomic analysis.

## Results and Discussion

### Capture and Sequencing

We sequenced the exomes of nine chimpanzees (CM), two crab-eating macaques (CE) and eight Japanese macaques (JP). Exonic sequences were enriched with the Agilent SureSelect all exon capture array (Human All Exon V1 for Human, CM and CE and Human All Exon V2 for JP)(Santa Clara, CA), targeting ∼38 Mb (∼46 Mb for JP) of DNA in nearly ∼18,000 human consensus coding DNA sequences (CCDS). Sequencing was performed on an Illumina Hiseq2000 sequencer (San Diego, CA). The two human individuals (HM) were sequenced using the same workflow. The human genome [hg19, UCSC] was used as the reference for alignment of all sequenced individuals, since it enabled consistent comparisons for all individuals using the same coordinate system. All reads were aligned using SOAPaligner [25] with a gap-free model and for SNP calling and coverage calculations. BWA [26] gap tolerant alignments were also used to detect short indels. For the non-human primates, we also mapped reads to their own or nearest reference genome (chimpanzees to panTro2, crab-eating macaques and Japanese macaque to rheMac2) to evaluate sequencing quality (Table S2).

### Evaluation and Comparison of Capture Performance

**Figure 1:**
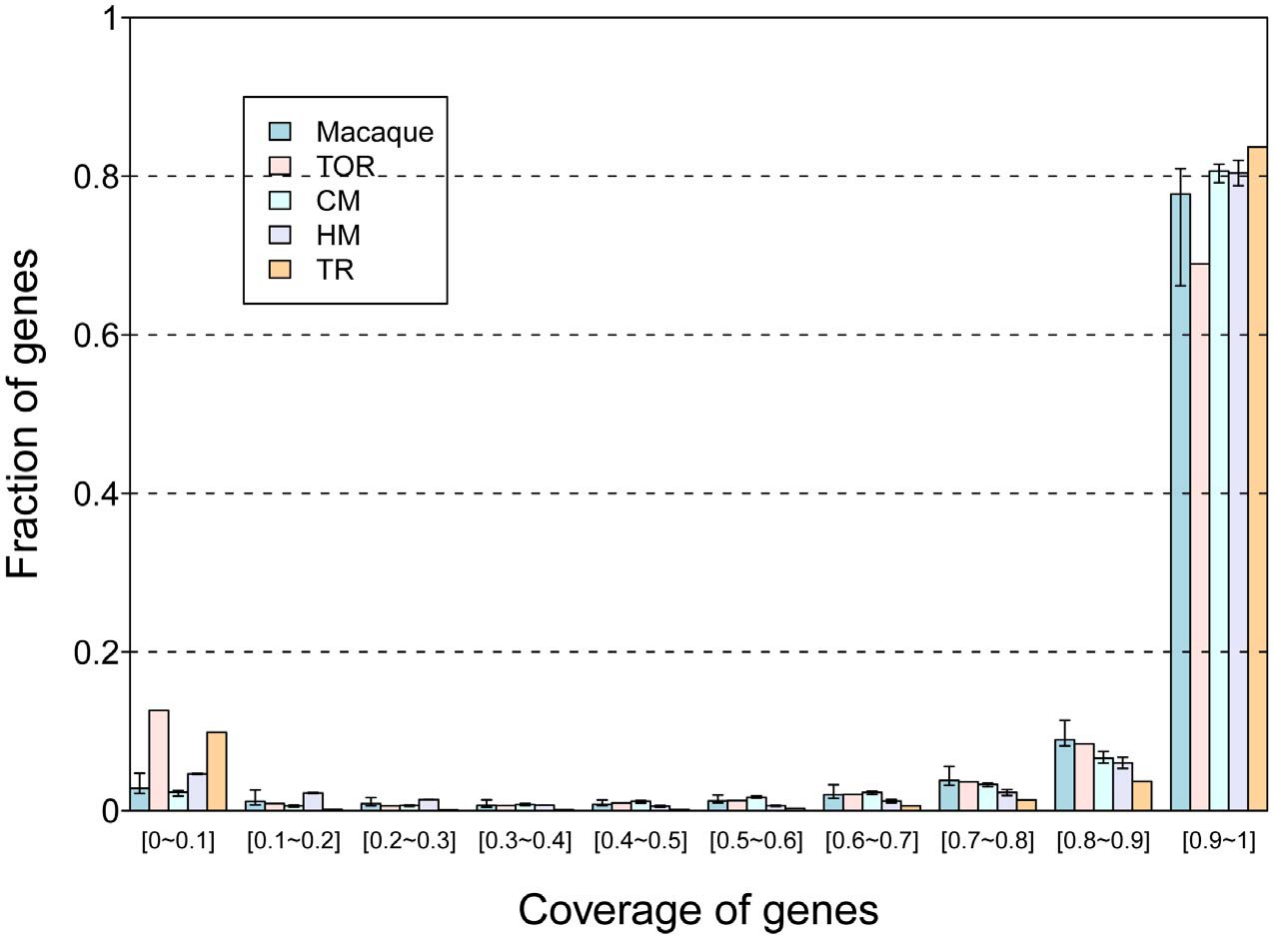
The profile of gene coverage. The distribution of coverage of 18,594 gene coding regions of human CCDS by theoretical target region, target orthologous region and sequencing data from 3 species is shown. The sequencing coverage of each gene was inferred from one individual in each species. Sequencing data showed that 80.40%–80.45% of genes were covered ≥90% for both humans and chimpanzees, and 77.76% for macaques. Coverage of macaque CCDSs was higher than target orthologous region. The coverage of human and chimpanzee genes was close when concerns target region, and higher for macaque compared to target orthologous region.

In order to perform unbiased evaluation, we compared the capture performance and sequencing quality in different species using equivalent metrics. Low quality reads and sequencing adaptor reads were filtered. Reads that mapped to the same chromosomal location and also had the same orientation were identified as duplicated reads. The one with highest mean quality score was retained and used in the analysis. The exomes of all 21 individuals were sequenced with a mean depth ≥28 fold (Table 1) on the array design target region (TR). Coverage of the TR ranged from 91.06% to 97.73%, with 67.59% to 81.33% of the sites in the TR covered by more than 10 reads. A gene-by-gene coding region coverage statistic was implemented for both theoretical TR and by actual sequence data. The theoretical analysis found that 83.68% (15559/18594) of CCDS genes’ coding regions should have ≥90% covered by TR (Table 2). The recovered sequence data showed that 80.40%–80.65% of the genes were covered by ≥90% of sequence reads for humans and chimpanzees, and by 77.76% for macaques (Figure 1). Since coverage of chimpanzee CCDS was nearly equivalent to that of humans, we primarily focused on the macaques for our additional analysis. When mapping to the closest reference genome, the mapping rate was ≥84% in all 21 individuals, indicating a high quality sequencing data.

**Table 1:** Data production.

	CE1	CE2	JP (mean ± s.d.)	CM (mean ± s.d.)	HM1	HM2
Target region (Mb)	37.63	37.63	45.88	37.63	37.63	37.63
# of clean reads(Mb)	31.71	51.12	98.43±8.24	27.55±2.75	25.56	25.56
Reads mapped to humangenome(Mb)	18.12	28.05	55.68±6.07	23.54±2.30	23.58	23.35
(fraction of clean reads%)	57.13	54.86	56.48±2.06	85.46±1.06	92.26	91.37
Reads mapped to human genomeafter filter duplication(Mb)	17.66	27.30	48.44±4.93	22.22±2.23	22.74	22.42
(fraction of clean reads%)	55.69	53.40	49.19±2.56	80.67±1.68	88.97	87.72
Reads mapped to own genome(Mb)	27.02	45.92	88.22±8.09	24.22±2.15	23.58	23.35
(fraction of clean reads%)	85.20	89.83	89.58±1.19	88.01±1.79	92.26	91.37
Reads mapped to target region(Mb)	14.26	21.04	88.22±8.09	16.67±1.71	17.08	17.35
(fraction of clean reads%)	44.96	41.17	40.66±2.95	60.51±1.60	66.82	67.89
Mean depth of target region	30.72	45.77	70±8.52	35.03±3.40	35.41	36.14
Coverage of target region(%)	91.06	92.70	93.29±0.14	97.16±0.18	97.08	97.73
Capture specificity	52.77	45.83	45.36±2.84	68.78±2.21	72.43	74.30
Fraction of target covered > = 4X(%)	81.14	83.67	87.03±0.40	91.06±0.83	91.30	92.27
Fraction of target covered> = 10X(%)	67.59	72.32	78.80±0.86	78.41±2.38	79.09	79.91
Rate of nucleotide mismatch (%)	2.90	2.76	2.76±0.07	1.03±0.05	0.44	0.48

Summary of captured target sequence coverage for each non-human primate exome and two human exomes. The total size of the captured target is 37,627,322 bp for CE and 45,880,359 bp for JP. Each exome was compared to the human reference genome. Listed for each exome are the details of captured data including alignment, depth and coverage of target region. The exomes of all twenty one individuals were sequenced with a mean depth ≥28 fold on array designed target region (TR). Coverage of target region ranged from 91.06% to 97.73%, and 67.59% to 81.33% of sites in the target region were covered by more than 10 reads. Non-human primates were aligned to their own or closest genomes (rheMac2 was used as reference genome for crab-eating cynomolgus and Japanese macaque) to evaluate the capture efficiency.

**Table 2:** Comparison of different scanning regions and theoretical coverage of genes.

	Target Region	Target Orthologous Region	Target Orthologous Region &depth≥10
Length(bp)	37,627,322	35,163,761	17,067,287
Fraction of raw Target Region	100.00%	93.45%	45.36%
Coverage of CCDS genes	86.21%	80.63%	44.93%

Summary of theoretical analysis for target region, target orthologous region and target orthologous region & depth≥10. Target orthologous region was the region we used to assess for capture efficiency and the target orthologous region & depth≥10 was used for SNP detection. 18,594 CCDS genes were used to analyze the theoretical coverage.

To further consider the exon sequence recovery, we used pairwise blastz alignment result of hg19/rheMac2 (download from UCSC genome browser) to evaluate the orthologous level between human and rhesus macaque in the TR. Evaluating a fragment from the target region of the human reference genome alignment to the Indian macaque reference genome, we calculated an orthologous score (OS) for every position and separated them into three levels: OS = 0, no alignment, unable to be captured theoretically; OS = 1, only one hit, high level of 1∶1 orthologous coverage; OS = 2, multiple hits at different locations in the macaque reference, suggesting misalignment and potential for calling false polymorphisms (Table 3). We defined the region where OS = 1 as target orthologous region (TOR) for macaques and only used data in those regions in the following analysis. The region contains 93.5% of the initial target region. In TOR, 12,817 genes theoretically could be ≥90% covered (Table 2) and the actual sequence data showed that 88.86% (11389/12817) of them were ≥90% covered (Table S1). Thus based upon the TOR analysis, the coverage was close to the expected.

**Table 3:** Distribution of orthologous score (OS) for macaques.

	OS = 0	OS = 1	OS = 2
Length	1.78 M	35.16 M	0.68 M
Percent of target region	4.73%	93.45%	1.82%

A fragment from the target region of the human reference genome was aligned to the Indian macaque reference genome. The OS was calculated for every site and classified into three possible levels: OS = 0, no alignment hit, unable to be captured theoretically; OS = 1, only one hit, high level of 1∶1 orthologous, well covered; OS = 2, multiple hits at different locations in the macaque reference, would give rise to misalignment and potentially false polymorphisms.

In assessing capture specificity, we defined the ratio of the reads aligned to a target region to the reads mapped to the species’ closest reference genome. We found that capture specificity ranged from 40% to 74% (Table 1) and decreased as a function of sequence divergence. The percentage of clean reads that mapped to the TR for macaque were much less than that of human (∼35% vs. ∼68%).

**Figure 2:**
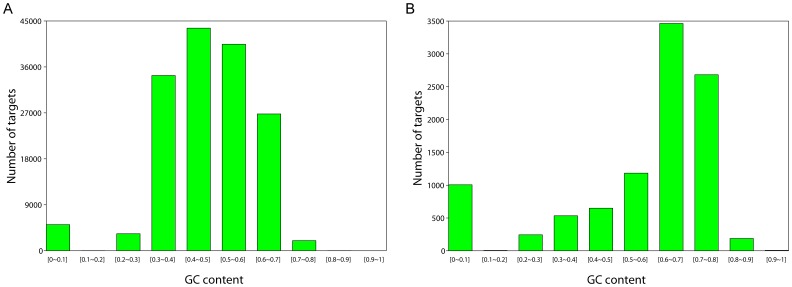
GC distribution of captured and un-captured targets by CE1. Sequenced CE1 exome was compared with its closest reference genome (rheMac2) to examine the influence of GC content on coverage. (A) Captured targets were defined as targets with more than 50% of bases covered at least by one read. The captured targets were mainly of moderate GC content. (B) Uncaptured targets were less than a half of the bases coverd by at least one read. The uncaptured targets were dominated by high GC content, except for a small portion with low GC content.

**Figure 3:**
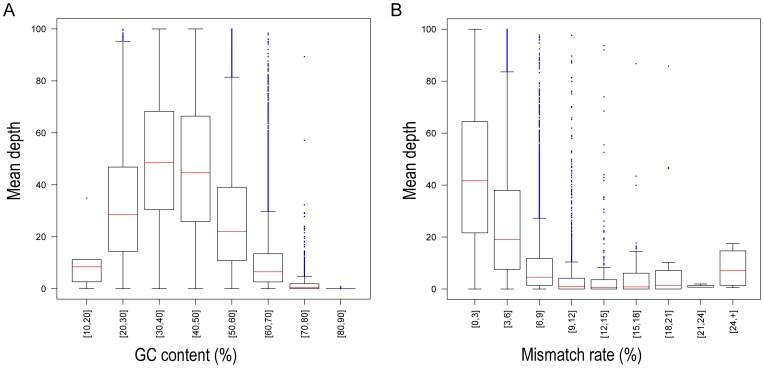
Average sequencing depth of captured targets versus human reference and rhesus macaque reference mismatch. Mismatch rate of each target region was calculated by comparing human reference and rhesus macaque references respectively. (A) The X-axis identifies the mismatch rate region, and the Y-axis identifies the mean depth of target region, as calculated from CE2. As the mismatch rate increases, the mean depth decreases. (B) Average sequencing depth of captured targets versus each target GC content. Targets with extreme GC content were poorly captured; the mean depth was lower compared with moderate GC content.

In order to further investigate the influence of specific genomic features on capture efficiency, we evaluated how targets that failed to be captured differed from targets that were successfully captured (more than 50% of bases covered at least by one read) (Table 4A and 4B). We found that failed targets generally had more nucleotide differences from human (6.95% vs. 3.29%), higher percentage of indels (1.93% vs. 0.64%) and a higher GC content (57% vs. 47%) in macaques. More detailed inspection of GC content revealed that the total GC content influence capture rate over a broad range (Figure 2 A and 2B). Target regions with moderate GC content (30%–50%) yielded higher coverage rates than regions with either high GC or low GC content (Figure 3A). This finding is likely the result of the methods employed for the exon-capture, which included use of annealing temperatures that were optimize for the binding of target sequences with a moderate GC content.

**Table 4:** Genomic features of captured and not captured targets.

(A) Genomic features of captured targets					
	# of targets	# of bases	mismatch	Indel bases	GC
CE1	155,610	35,682,723	3.07%	0.55%	47.13%
CE2	157,062	36,002,143	3.10%	0.56%	47.16%
JP1	185,636	44,284,360	3.28%	0.63%	47.26%
JP2	186,122	44,395,960	3.29%	0.64%	47.30%
JP3	185,628	44,295,992	3.28%	0.63%	47.26%
JP4	185,584	44,280,953	3.28%	0.63%	47.24%
JP5	185,481	44,251,493	3.28%	0.63%	47.24%
JP6	185,540	44,256,348	3.28%	0.63%	47.23%
JP7	186,066	44,364,842	3.29%	0.64%	47.28%
JP8	185,812	44,305,013	3.28%	0.64%	47.26%
**(B) Genomic features of not captured targets**					
	**# of targets**	**# of bases**	**mismatch**	**Indel bases**	**GC**
CE1	9,961	1,944,599	5.85%	1.47%	56.29%
CE2	8,509	1,625,179	5.90%	1.47%	57.15%
JP1	8,381	1,595,999	6.85%	1.87%	53.84%
JP2	7,895	1,484,399	6.86%	1.89%	53.27%
JP3	8,389	1,584,367	6.83%	1.88%	53.84%
JP4	8,433	1,599,406	6.82%	1.88%	54.26%
JP5	8,536	1,628,866	6.80%	1.85%	54.13%
JP6	8,477	1,624,011	6.86%	1.87%	54.27%
JP7	7,951	1,515,517	6.95%	1.93%	53.70%
JP8	8,205	1,575,346	6.92%	1.88%	53.99%

We examined the following genomic features for the 165,571 (for CE) and 194,017 (for JP) human targets with best reciprocal orthologs in the Indian macaque genome: the number of nucleotide differences between human and macaque, the number of indel bases between human and macaque and the GC content. (A) A target was considered “captured” if more than half of the human targeted bases were covered by at least one sequence read. (B) Otherwise, the target was not captured.

Finally, we calculated the mismatch rate with the human and rhesus macaque reference genomes and correlated that with the average sequencing depth for all 158,852 autosomal targets. We found that sequencing depth decreased as sequence divergence increased (Figure 3B).

The remaining unrecovered TOR in macaques totaled 1.74 Mb, accounted for by three potential factors: 1) there were ∼0.22 Mb sites absent from both HM and CE in this capture array design; 2) the rhesus macaque genome was used to identify the TOR, and sequence divergence of the CE and JP genomes could have limited exon capture potential; 3) the amount of sequence, in combination with the reduced efficiency of capture, may have resulted in a biased representation of exon coverage.

We conclude that we can predict the capture performance using available pairwise alignment information for those species that have their own/related reference genome. We observed TOR regions for Orangutan [ponAbe2] and Marmoset [calJac3] had a percentage of 92.26% and 88.80% separately in TR, using the same analysis with the IR genome. This demonstrates a potential utility of human based chip among these species. On the other hand for species without reference genomes, but which are very closely related to humans, exon capture should be sufficiently efficient to make sequencing the majority of the exome, as well as genotyping each individual, feasible.

### Variation Discovery and Analysis

The use of exome sequencing for mutation discovery in human studies is critically dependent on the accurate identification of DNA polymorphisms and genotypes. We were interested in whether human exon capture technology would also enable the accurate detection of variants in non-human primates. Using the reference genomes of humans and non-human primates, it was possible to identify both variations between species and within species.

We used SOAPsnp [27] for calling genotypes. In order to directly compare different individuals, we only called genotypes in TORs where every individual had a read depth of ≥10X and had high quality reads. The qualifying regions totaled 17.07 Mb, or 45.36% of the original TR (Table 2). In total, 79,704 ∼311,477 single base variants were detected within non-human primates (compared with human reference) and 15,013∼27,391 of them were candidate intra-species polymorphisms (Table 5A).

**Table 5:** (A) Comparison of sequencing based point variants and variants between references. (B) Coding SNP summary; piN refers to X and piS refers to Y. dN indicates X and dS indicates Y.

(A) Comparison of sequencing based point variants and variants between references.								
	Total SNPs	consistent		inconsistent		non-overlap	candidate intra-species polymorphism	consistent rate
		hom	het	hom	het			
JP1	311,252	287,901	4,067	532	37	18,715	23,351	99.81%
JP2	310,564	287,724	3,935	536	42	18,327	22,840	99.80%
JP3	310,948	288,188	3,927	524	45	18,264	22,760	99.81%
JP4	310,935	287,036	4,115	521	47	19,216	23,899	99.81%
JP5	311,477	287,427	4,195	519	40	19,296	24,050	99.81%
JP6	310,847	287,096	4,131	525	36	19,059	23,751	99.81%
JP7	310,132	285,929	4,181	528	45	19,449	24,203	99.80%
JP8	311,362	287,570	4,131	513	40	19,108	23,792	99.81%
CE1	306,534	279,641	5,929	543	49	20,372	26,893	99.79%
CE2	303,992	276,601	5,910	517	44	20,920	27,391	99.80%
CM1	80,882	64,328	2,398	67	18	14,071	16,554	99.87%
CM2	80,571	64,042	2,446	71	21	13,991	16,529	99.86%
CM3	80,721	64,433	2,379	68	19	13,822	16,288	99.87%
CM4	80,061	63,718	2,392	70	15	13,866	16,343	99.87%
CM5	80,895	64,498	2,271	74	18	14,034	16,397	99.86%
CM6	80,737	64,230	2,391	68	16	14,032	16,507	99.87%
CM7	80,381	64,034	2,407	77	16	13,847	16,347	99.86%
CM8	79,704	64,691	2,047	71	16	12,879	15,013	99.87%
CM9	80,500	64,228	2,346	70	17	13,839	16,272	99.87%
**(B) Coding SNP summary**								
	**Non synonymous** **SNPs**	**Synonymous** **SNPs**	**Heterozygotes**	**piN/piS**	**Non synonymous divergence**	**Synonymous divergence**	**dn/ds**	**Neutrality Index**
JP1	8,172	10,757	13,096	0.7597	60,162	155,835	0.3861	1.9678
JP2	7,948	10,503	12,651	0.7567	60,089	155,702	0.3859	1.9608
JP3	7,896	10,513	12,638	0.7511	60,224	156,046	0.3859	1.9461
JP4	8,429	10,922	13,662	0.7717	59,936	155,274	0.386	1.9993
JP5	8,502	10,991	13,827	0.7735	59,995	155,564	0.3857	2.0058
JP6	8,382	10,876	13,563	0.7707	60,022	155,398	0.3862	1.9953
JP7	8,603	11,053	14,014	0.7783	59,738	154,756	0.386	2.0164
JP8	8,348	10,913	13,643	0.765	60,104	155,724	0.386	1.9819
CE1	7,477	13,753	16,904	0.5437	58,382	151,782	0.3846	1.4134
CE2	7,850	13,719	17,330	0.5722	57,596	149,784	0.3845	1.4881
CM1	5,207	7,449	9,975	0.699	16,421	31,766	0.5169	1.3522
CM2	5,292	7,492	10,240	0.7064	16,301	31,622	0.5155	1.3702
CM3	5,119	7,305	9,714	0.7008	16,399	31,852	0.5148	1.3611
CM4	5,191	7,477	10,044	0.6943	16,246	31,466	0.5163	1.3447
CM5	5,211	7,325	9,698	0.7114	16,461	31,879	0.5164	1.3777
CM6	5,179	7,389	9,885	0.7009	16,359	31,759	0.5151	1.3607
CM7	5,176	7,417	9,833	0.6979	16,296	31,670	0.5146	1.3562
CM8	4,763	6,789	8,287	0.7016	16,511	31,975	0.5164	1.3587
CM9	5,272	7,329	9,934	0.7193	16,342	31,719	0.5152	1.3962
HM1	2,944	4,024	4,282	0.7316				
HM2	2,957	4,092	4,310	0.7226				

We considered the possibility of cross DNA contamination contributing to the SNPs called. Though we used care in the DNA extraction, capture, library construction and sequencing procedures, we nonetheless evaluated whether the derived exome sequence contained evidence of human DNA contamination. We compared all exome sequencing data to the closest reference sequence. We found that the exome sequences and closest reference sequences exceeded a 99% match in all 19 non-human primate individuals (Table 5A). These results indicate that the sequence data used for genotype calling aligned most closely with the reference genome and did not suggest the presence of contaminating human DNA.

The abundance of polymorphisms discovered in coding and flanking regions enabled us to explore the potential evolutionary history of these species on a population scale. We found the macaques had the highest level of heterozygosity, followed by the chimpanzee. These data suggest that the macaques have a higher effective population size than chimpanzees, which in turn have a higher effective size than humans (Table 5B). In all species the ratio of nonsynonymous to synonymous SNPs was larger than the ratio of nonsynonymous to synonymous differences (Neutrality index, Table 5B) This is consistent with previous studies of effective population sizes in these species, [13], [17] suggesting that nonsynonymous deleterious alleles are the subject of selection pressure within each species.

**Figure 4:**
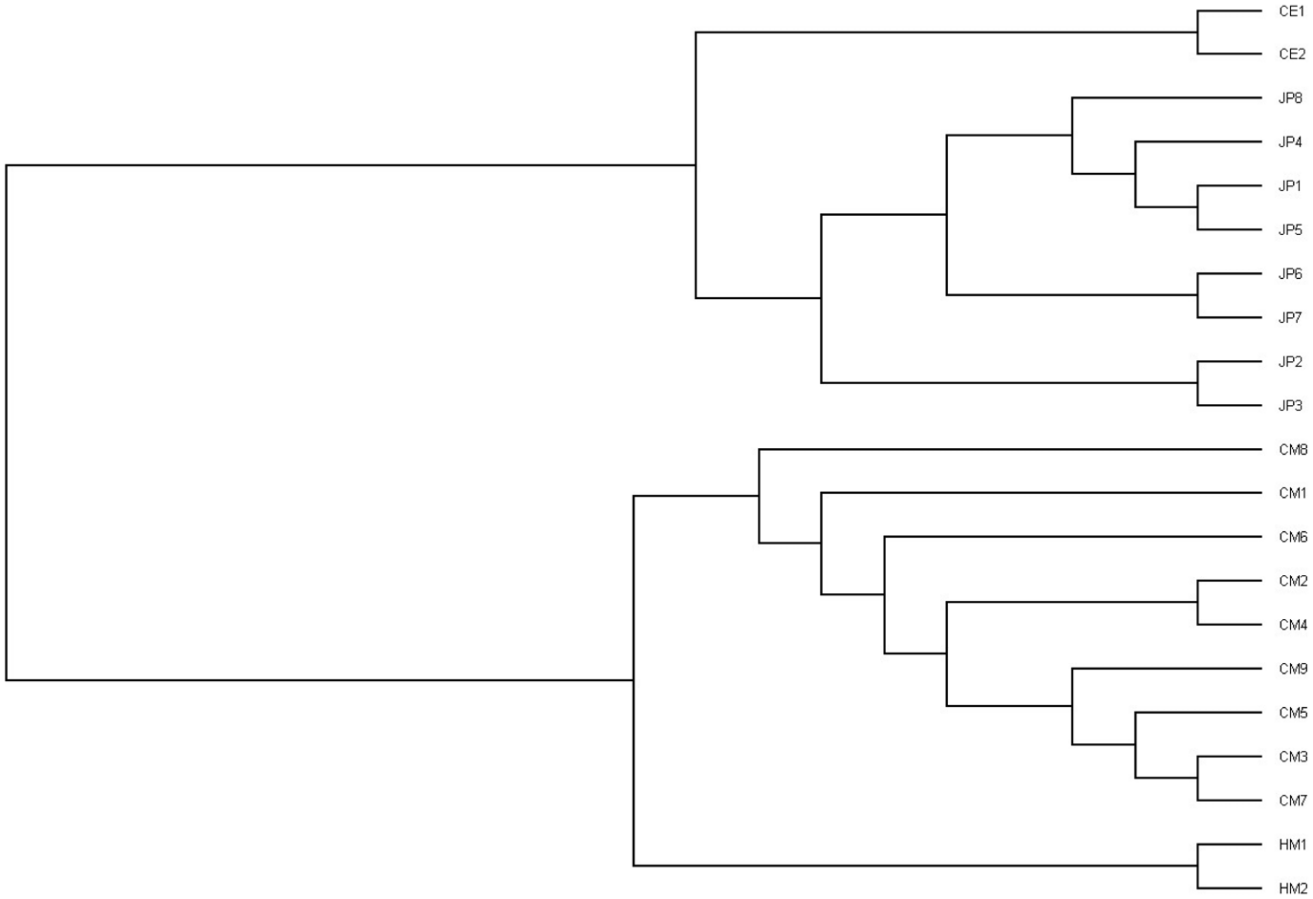
Phylogenetic tree. Evolutionary distance in human, chimpanzee and macaque lineages, showing the inferred evolutionary relationships among 21 samples based upon similarities and differences in their genetic characteristics. The taxa joined together in the tree are implied to have descended from a common ancestor.

**Figure 5:**
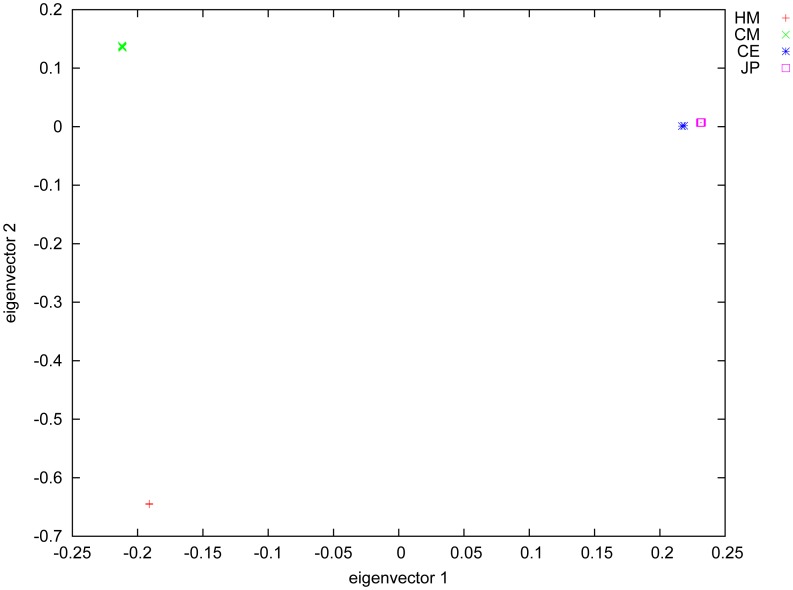
Principle Component Analysis. Principal components analysis plot of 9 chimpanzees, 2 crab-eating macaques, 8 Japanese macaques and 2 human samples. Chimpanzees and humans cluster well within their own group, while crab-eating and Japanese macaques spread away are in relatively close proximity to each other, indicating recent genetic divergence between them.

Since the nineteen non-human primate exomes included three different species, we used the polymorphism data to evaluate the evolutionary relationships among individuals. We constructed a phylogenetic tree (Figure 4) using the neighbor-joining (NJ) method. The phylogenetic tree is consistent with the ancestry analysis shown in previous genome studies [17], [18], as well as with results based upon microsatellite markers [1]. Principal component analysis (PCA) also showed that the individuals in each species were genetically most similar (Figure 5).

**Figure 6:**
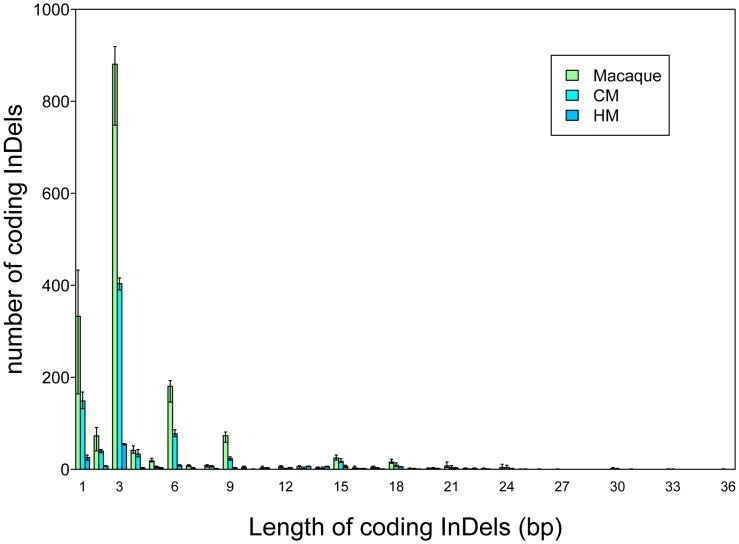
The length distribution of coding indels for each individual. 140∼1,883 coding indels were called in each individual, and 54.61%∼77.32% of them were 3 bp in length. All indels were relative to the human reference genome.

We also identified short insertions and deletions (indels) in the exons, which are likely to be functionally important and may contribute to species divergence (Table S3). Here we mapped the reads from each individual to the human reference sequence using BWA and Samtools [28] to identify indels (see methods for details). In total, 140∼1,883 (range for all individuals) human-primate coding indels were discovered in each individual (Table 6), and 54.61%∼77.32% of them were 3 base pairs in length. The distribution of indel lengths in the coding regions is consistent with a previous study [29] across all of the 21 exomes (Figure 6), reflecting the evolutionary pressure to preserve intact reading frames.

**Table 6:** Indel summary.

	CE1	CE2	JP (mean ± s.d.)	CM (mean ± s.d.)	HM1	HM2
Total number of indels	13,890	17,417	17,482.8±475.4	6,693.7±334.3	773	697
Ins-coding	698	910	1,072.4±40.3	427.6±17.6	93	84
Del-coding	607	698	696.5±17.6	360.3±8.4	59	56
Splice site	885	1,078	833.1±17.4	303.7±49.5	45	33
Intron	10,813	13,615	13,771.6±398.6	5,079.8±267.4	518	465
5′ UTRs	256	341	344.5±8.2	190.3±6.3	22	29
3′ UTRs	579	709	701.4±8.3	280.9±14.8	30	25
Intergenic	52	66	63.3±2.3	51.1±2.3	6	5
Total insertion	7,460	9,589	11,127.4±321.8	3,673.7±177.5	437	395
Total deletion	6,430	7,828	6355.4±155.8	3,020±177.6	336	302
Heterozygous indels	378	536	295.0±19.3	452.4±60.2	280	240
Homozygous indels	13,512	16,881	17,187.8±457.9	6,241.2±277.5	493	457

Each exome from 21 individuals was aligned to the human reference genome for indel identification.

We carried out Sanger sequencing for validation on a randomly selected 41 SNPs and 18 indels in two exome sequenced CE individuals. The validation rate was 85.4% for SNPs and 84.2% for indels (Table S4), indicating a high accuracy rate.

## Discussion

Here we evaluated the efficiency of exome capture and sequencing in non-human primates using a human capture array. We determined that although capture specificity decreased as sequence divergence increased, it is still a viable option for crab-eating macaque, Japanese macaque and Chimpanzee exome enrichment. We also identified inter-species and intra-species variation by comparing recovered sequences to the rhesus macaque reference genome, and then validated the call accuracy using Sanger resequencing.

One limitation of this method is the inability to detecting large structural variations, such as large indels, inversions and copy number variations (CNV). In addition, the particular human exon capture design may miss some important 5′ and 3′ noncoding as well as small RNAs. Further, a subset of the genomic target regions were not recovered.

Despite the limitations, use of human exome-enrichment has some distinct advantages. We were able to efficiently capture gene coding region for both species with reference genomes, as well as those without a species-specific reference genome. Applications of this method could enable the efficient, large scale exon-sequencing of non-human primates, potentially identifying DNA variants of relevance to human disease, and guiding animal model development and translational research. Finally, exome-capture approaches could expedite the comparative genomic analysis of non-human primate species, for example between gorilla, orangutan, gibbons, baboon, and even New World monkeys, providing insight to the evolution of the human genome.

The nineteen non-human primate exomes presented here also highlight the degree of variation existing between these widely used non-human primate animal models. The abundant genetic diversity evident in individual primates from distinct geographic populations is of direct interest to primatology, medical research, population genetics and phylogeographic studies.

## Materials and Methods

### Ethics Statement

Blood samples of 2 Han Chinese individuals were collected from Anhui Medical University. We have obtained ethics approval for our study from the Ethical Committee of Anhui Medical University. We have also obtained informed written consent from all participants involved in this study. The authors of this paper did not collect the blood samples themselves; all non-human primate blood samples used in this study were initially collected for routine health check purposes.

Samples from nine wild born, unrelated chimpanzees (*Pan troglodytes schweinfurthii)* were collected at the Ngamba Island Chimpanzee Sanctuary in Uganda. The samples were taken during routine health checks by the sanctuary veterinarians and were exported under CITES export permit Sn.UG 002249. The Ugandan Wildlife Authorities and Uganda National Council for Science and Technology approved the research that resulted in the CITES export permission.

Two adult cynomolgus macaque *(Macaca fascicularis)* were individually housed in cages with the size of 70 cm×70 cm×80 cm by the South-China Primate Research & Development Center (Guangdong Landau Biotechnology Co., China) under the temperature of, between 16°C and 29°C and 50∼80% relative air humidity, with four air changes per hour and a 12 h light:12 h dark cycle. Animals were fed apples (100 g/day each) and monkey Chow (Feed Research Institute, Guangzhou, Guangdong) twice daily and allowed free access to water. The blood samples were collected during routine veterinary health checks. Guangdong Landau Biotechnology Co. is accredited by the Association for Assessment and Accreditation of Laboratory Animal Care International (AAALAC). The macaques were originally exported from Kampuchea under CITES export permit Sn. IC 0381. All macaque experiments were subject to approval and surveillance by the Institutional Animal Care and Use Committee of Guangdong Landau Biotechnology Co., Ltd. Genomic DNA was extracted from blood samples using the Nucleon kit (TaKaRa, Japan). Blood derived DNA was used to minimize somatic and cell-line derived false positives.

Eight Japanese macaques (*Macaca fuscata)* were born and raised at the Oregon National Primate Research Center (ONPRC); all animal procedures were approved by the ONPRC Institutional Animal Care and Use. Committee and conformed to the NIH guidelines on the ethical care and use of animals in research. The selected animals were members of captive breeding group that lived within in a 2 acre, outdoor corral, and consumed Primate Diet no. 5000, (Lab Diet, Richmond, IN). Blood samples were collected during routine veterinary care, by venipuncture and collection into an EDTA vacutainer. Genomic DNA was isolated using the Wizard DNA Extraction Kit (Promega, Inc.).

### Exon Capture and Sequencing

The qualified genomic DNA samples were randomly fragmented by Covaris and the sizes of the library fragments were distributed between 150 to 200 bp. Adapters were then ligated to both ends of the resulting fragments. The adapter-ligated templates were purified by the Agencourt AMPure SPRI beads and fragments with insert size of about 250 bp were excised. Extracted DNA was amplified by ligation-mediated PCR (LM-PCR), purified, and hybridized to the SureSelect Biotinylated RNA Library (BAITS) for enrichment. Hybridized fragments were bound to the strepavidin beads whereas non-hybridized fragments were washed out after 24 h. Captured LM-PCR products were subjected to Agilent 2100 Bioanalyzer to estimate the magnitude of enrichment.

Each captured library was loaded on the Illumina Hiseq2000 platform for high-throughput sequencing to the desired average sequencing depth (The exomes of all 21 individuals were sequenced with a mean depth ≥28 fold on the array design target region). Raw image files were processed by Illumina basecalling Software 1.7 for base-calling with default parameters and the sequences of each individual were generated as 90 bp pair-end reads.

### Read Mapping and Genotype Calling

SOAPaligner (soap 2.21) was used to align reads to the human reference genome (hg19) allowing a maximum of 3 mismatches per 90 bp read. Full SOAP parameters were: -a -b -D -o -2 -t -v 3 -l 35 -s 40 -m 0 -x 500 -p 4 -r 1 (-v 5 for macaques). Reads that aligned to the designed target region (TR) were collected for genotype calling and subsequent analysis.

Based on SOAP alignment results, the software SOAPsnp was used to call genotypes. The following parameters were set: -r 0.0005 -e 0.001 -t -u -2 -i -d -o -M -L 90 -s -T(http://soap.genomics.org.cn/ for details).

Raw data has NCBI Short Read Archive accession no. SRA038809.

### SNP Identification

Single base differences between human and non-human primates references (ref-ref variants) were identified from pairwise blastz alignments result. *.axt files for Human/Chimp (panTro2) and Human/Rhesus (rheMac2) were downloaded from UCSC Genome Browser (http://hgdownload.cse.ucsc.edu/downloads.html#human).

Raw point variants from exome sequencing data of each individual were extract from SOAPsnp generated genotype with filter criteria Q20 and depth ≥10. Homozygous variants consistent with ref-ref variants were defined as interspecies variants. Remaining raw point variants were considered to be potential intraspecies polymorphisms.

### Targeted Orthologous Region (TOR)

Orthologous score (OS) for every site in TR was calculated with pairwise blastz alignments result. The *.axt files for Human/Chimp, Chimp/Human (panTro2) and Human/Rhesus, Rhesus/Human (rheMac2) were downloaded from UCSC Genome Browser (http://hgdownload.cse.ucsc.edu/downloads.html#human).

Alignment of fragments from the TR between human and non-human primates were used to calculate OS. Alignment hits count = 0, OS = 0; alignment hits count = 1, OS = 1; alignment hits count≥2, OS = 2. Regions in TR where OS = 1 were defined as TOR.

### Phylogenetic Tree

We use high quality genotypes on chromosome 10 (TOR, depth≥10 and consensus quality≥20 in every individual) generated by SOAPsnp to build a phylogenetic tree with the program TreeBeST (treebest-1.9.2, http://treesoft.sourceforge.net/treebest.shtml; L.Heng, A.J. Vilella, E.Birney, and R.Durbin, in prep) with the model of neighbour-joining tree, SDI, rooting (threshold: nj -b 500).

### Principal Component Analysis (PCA)

We use high quality genotypes on chromosome 10 (TOR, depth≥10 and individuals whose quality score under 20 were discarded) generated by SOAPsnp to perform principal component analysis (PCA) using EIGENSOFT3.0 (parameter: -i all.evec -c 1∶2 -p HM:CM:CE:JP -x).

### Identification of Insertions and Deletions

Gap tolerant alignments to human reference (hg19) were used to call indels with program BWA using parameters: aln -o 1 -e 63 -i 15 -L -l 31 -k 2 -t 4. Insertions and deletions (indels) were identified using samtools, with the command lines as following:

samtools mpileup -ugf ref.fa -b bam.list | bcftools view -bvcg - > var.raw.bcf

bcftools view var.raw.bcf | vcfutils.pl varFilter -D10000> var.flt.vcf.

Filter criteria were ≥3 reads support and number of indel supported reads ≥30% of all reads mapped to the genomic position. Indels were called as heterozygous if the indel supported reads were 30–70% of all reads at that position, and homozygous if they were greater than 70%.

### Validation of SNP and Indel Variants

Forty one SNPs and 18 indels were selected randomly for validation of SNPs and indels. The selected SNP and indels were genotyped by PCR and Sanger sequencing. Primers for each selected SNP and indel were designed based on the IR genome; the detail sequences of each primer pairs were supplied in Table S4. The polymerase chain reaction (PCR) was performed in a final volume of 50 ul with 30 cycles at 94°C for 30 s, annealing temperature (Table S4) for 30 s, and 72°C for 30 s. The PCR product was purified and sequenced by BGI (BGI-Shenzhen, Shenzhen 518083, China).

## Supporting Information

Table S1 **Coverage of targeted orthologous genes.** Coverage of 18,594 gene coding regions of human CCDS by theoretical target region, target orthologous region and sequencing data from 3 species.
(RAR)

Table S2 **Data production.** Summary of captured target sequence coverage for each non-human primate exome and two human exomes.
(XLSX)

Table S3 **Indels.** Indels of 21 individuals identified through aligning the exomes to the human reference genome.
(XLSX)

Table S4 **Sanger sequencing validation result of SNP and Indels.**
(XLS)
