# Correction: An Effort to Use Human-Based Exome Capture Methods to Analyze Chimpanzee and Macaque Exomes

**DOI:** 10.1371/annotation/450f85d8-03c6-4bd4-aa8e-b5b5894b4593

**Published:** 2012-09-28

**Authors:** Xin Jin, Mingze He, Betsy Ferguson, Yuhuan Meng, Limei Ouyang, Jingjing Ren, Thomas Mailund, Fei Sun, Liangdan Sun, Juan Shen, Min Zhuo, Li Song, Jufang Wang, Fei Ling, Yuqi Zhu, Christina Hvilsom, Hans Siegismund, Xiaoming Liu, Zhuolin Gong, Fang Ji, Xinzhong Wang, Boqing Liu, Yu Zhang, Jianguo Hou, Jing Wang, Hua Zhao, Yanyi Wang, Xiaodong Fang, Guojie Zhang, Jian Wang, Xuejun Zhang, Mikkel H. Schierup, Hongli Du, Jun Wang, Xiaoning Wang

There was a formatting error in Table 5 in the PDF. On page 8, an incorrect heading was given for the second page of Table 5. "(A) Comparison of sequencing based point variants and variants between references" should be "(B) Coding SNP summary". 

